# A Hopf physical reservoir computer

**DOI:** 10.1038/s41598-021-98982-x

**Published:** 2021-09-30

**Authors:** Md Raf E Ul Shougat, XiaoFu Li, Tushar Mollik, Edmon Perkins

**Affiliations:** LAB2701: Nonlinear Dynamics Laboratory, Department of Mechanical and Aerospace Engineering, North Carolina State University, Raleigh, NC 27695 USA

**Keywords:** Mathematics and computing, Engineering, Mechanical engineering

## Abstract

Physical reservoir computing utilizes a physical system as a computational resource. This nontraditional computing technique can be computationally powerful, without the need of costly training. Here, a Hopf oscillator is implemented as a reservoir computer by using a node-based architecture; however, this implementation does not use delayed feedback lines. This reservoir computer is still powerful, but it is considerably simpler and cheaper to implement as a physical Hopf oscillator. A non-periodic stochastic masking procedure is applied for this reservoir computer following the time multiplexing method. Due to the presence of noise, the Euler–Maruyama method is used to simulate the resulting stochastic differential equations that represent this reservoir computer. An analog electrical circuit is built to implement this Hopf oscillator reservoir computer experimentally. The information processing capability was tested numerically and experimentally by performing logical tasks, emulation tasks, and time series prediction tasks. This reservoir computer has several attractive features, including a simple design that is easy to implement, noise robustness, and a high computational ability for many different benchmark tasks. Since limit cycle oscillators model many physical systems, this architecture could be relatively easily applied in many contexts.

## Introduction

Reservoir computing (RC) is a bio-inspired, supervised machine-learning computational framework based on artificial recurrent neural networks (RNNs), which utilizes naturally emergent dynamics of a physical resource^1–6^. Conventional machine learning schemes use backpropagation through time^7^ to train an *entire* recurrent neural network. This method is computationally expensive, since all the weights of the network need to be updated to mimic a target function. *Echo state networks*^8^ and *liquid state machines*^9^ are two concepts that addressed this issue in the early 2000s. Reservoir computing merges these concepts. In reservoir computing, the neural network is formed from a set of coupled nonlinear nodes, where the network is divided into three parts: an input layer, the reservoir, and the readout layer. Unlike conventional RNNs, only the readout layer requires training by a simpler training algorithm, such as linear or ridge regression^10^. Thus, the RC architecture is much faster and more stable than conventional RNN methods, which is one of the key advantages of this information processing framework.

There are many real-world applications of reservoir computing, including bit-wise logical operations^11–13^, speech recognition^6^, handwritten digit recognition^14^, wireless communications^1^, complex and chaotic time series predictions^1,6,15–18^, image recognition^19^, emulation of nonlinear time series^4,10^, and morphological computation^20,21^. The echo state architecture of a reservoir allows the use of physical systems as reservoir computers, also known as *physical reservoir computers* (PRCs). Many physical systems have been shown to perform as PRCs, including an array of nonlinear mechanical oscillators^11,22,23^, soft robotic bodies^20,24–26^, tensegrity structures^21,27^, and origami structures^28,29^.

Importantly, quantum systems can be used as PRCs. The natural disordered quantum dynamics of an ensemble system was utilized to emulate nonlinear time series, including a chaotic system^30^. A Kerr nonlinear oscillator was used in sine wave phase estimation using its complex amplitudes as computational nodes^31^. Nuclear-magnetic-resonance spin-ensemble system was used for nonlinear dynamics emulation task by implementing spatial multiplexing approach to increase computational power^32^. Dissipative quantum dynamics was used to build a quantum reservoir computer (QRC) for nonlinear temporal tasks^33^.

Physical reservoir computers were initially constructed from only the coupled, real dynamic nodes. Later, a virtual node-based reservoir computing method was proposed by implementing a time multiplexing approach in which a delayed feedback was used as a single nonlinear dynamic node to perform computation^6^. This method simplifies the complexity of a reservoir built from an array of physical nonlinear nodes. This approach has been popularly used to construct physical reservoir computers for different tasks, such as an optoelectronic oscillator for optical information processing^34^, a photonics-based passive linear fiber reservoir for signal processing^35^, an FPGA implementation using a single autonomous Boolean logic element for pattern recognition^5^, time-delay reservoirs for forecasting of stochastic nonlinear time series^36^, a delayed Duffing silicon beam for parity tasks^12^, and a semiconductor laser with delayed optical feedback for nonlinear time series prediction^37^. These reservoirs used a delay line to create the necessary nodes for computation. A simpler approach can be taken by creating the nodes without the presence of any delay or feedback line^38^. This approach is studied less, though it makes the reservoir architecture much simpler.

Here, a Hopf oscillator is used as a physical reservoir. The Hopf oscillator can also be used as the building block for adaptive oscillators^39,40^, which can natively learn information without any training. The Hopf oscillator can exhibit limit cycle motion, which provides a source of memory by storing information in its dynamic states. Although a binary periodic masking function is popularly used for time-multiplexed reservoir^6,10,12^, noise can also be used as periodic mask^41^. In this paper, a Hopf oscillator PRC is constructed that uses a non-periodic stochastic mask. A Hopf oscillator physical reservoir computer is fabricated as an analog circuit, which is compared with Euler–Maruyama simulations^40,42,43^. This Hopf PRC can successfully complete benchmark machine learning tasks, including parity tasks, fundamental logic gate tasks^12^, nonlinear dynamic emulation tasks^4^, and various time series prediction tasks^44^. The information rate is used as the performance metric for logical tasks^11^, and the normalized mean square error (NMSE) is used for the emulation and time series tasks^4^.

The rest of the article is organized as follows. In “System equations for Hopf physical reservoir computer” section, the equations of motion for the stochastic Hopf oscillator PRC are presented. In “Mapping methodology” section, the methodology of mapping the oscillator’s dynamics to an information processing scheme is discussed for an example task by using the Euler–Maruyama simulation. The effects of the pseudo-period and the noise on computational ability are discussed in “Pseudo-period and noise” section. In “Analog circuit experiment” section, the analog circuit experiment is described. In “Benchmark tasks for Hopf PRC” section, different benchmark tasks are performed with the numerical and experimental Hopf PRC, which includes logic tasks, emulation tasks of time series, and prediction tasks. The concluding remarks are stated in “Concluding remarks” section.

## System equations for Hopf physical reservoir computer

**Table 1:** List of parameters, states, and functions.

Description	Nomenclature	Description	Nomenclature
First state	*x*	Second state	*y*
Input	*u*	Mask	*m*
Resonance constant	$\omega _0$	Amplitude of sinusoidal forcing	*A*
Frequency of sinusoidal forcing	$\Omega$	Phase of sinusoidal forcing	$\phi$
Parameter affecting limit cycle radius	$\mu$	Noise amplitude	$\sigma$
White Gaussian noise	${\dot{W}}$	Noise bias	$\beta$
Re-scaled *x* state	*X*	Identity matrix	*I*
Nodal state matrix	*L*	Target vector	*M*
Information rate	*R*	Pseudo-period	$T_p$
Output of PRC	*o*	Number of nodes	*N*

The equations of motion for the Hopf oscillator are^45^:

1 $$\begin{aligned} \begin{array}{ll} {\dot{x}} &{} = \big (\mu -(x^2+y^2) \big )x - \omega _0 y + A \sin (\Omega t+\phi ) \\ {\dot{y}} &{} = \big (\mu -(x^2+y^2) \big )y + \omega _0 x \end{array} \end{aligned}$$

For this Hopf oscillator, *x* and *y* are the first and second states, respectively, and the sinusoidal forcing is given by $A \sin (\Omega t+\phi )$. A list of the parameters is given in Table 1. The information is first encoded as an input, *u*(*t*), which will depend on the benchmark task being performed. The mask is defined by white Gaussian noise as:

2 $$\begin{aligned} m(t) = \sigma {\dot{W}} +\beta \end{aligned}$$

Here, $\sigma$ is the noise amplitude, ${\dot{W}}$ is white Gaussian noise, and $\beta$ is a positive bias. It should be noted that ${\dot{W}}$ does not exist, but its differential form, *dW*, does^46^.

To send information to the PRC to be processed, an external forcing function that contains the information signal, *u*(*t*), and the stochastic mask, *m*(*t*), is constructed as:

3 $$\begin{aligned} f(t) = 1 + u(t) m(t) \end{aligned}$$

This external forcing function is injected into both the amplitude of the sinusoidal forcing, *A*, and the parameter affecting the limit cycle radius, $\mu$. Including this force, the equations for the Hopf PRC are written as:

4 $$\begin{aligned} \begin{array}{ll} {\dot{x}} &{} = \Big (\mu f(t)-(x^2+y^2)\Big )x- \omega _0 y + A f(t) \sin (\Omega t+\phi ) \\ {\dot{y}} &{} = \Big (\mu f(t)-(x^2+y^2)\Big )y + \omega _0 x \end{array} \end{aligned}$$

## Mapping methodology

To use the dynamics of the Hopf oscillator as a physical reservoir computer, the dynamics must first be mapped. To describe this mapping, an exclusive OR (XOR) logical task is used as an example. In this section, the Hopf PRC is simulated using an Euler–Maruyama scheme, since the mask is stochastic^42^. Shannon’s information metric is used to quantify the performance of the reservoir when performing logical tasks, such as the XOR operation^11,43^.

**Figure 1:**
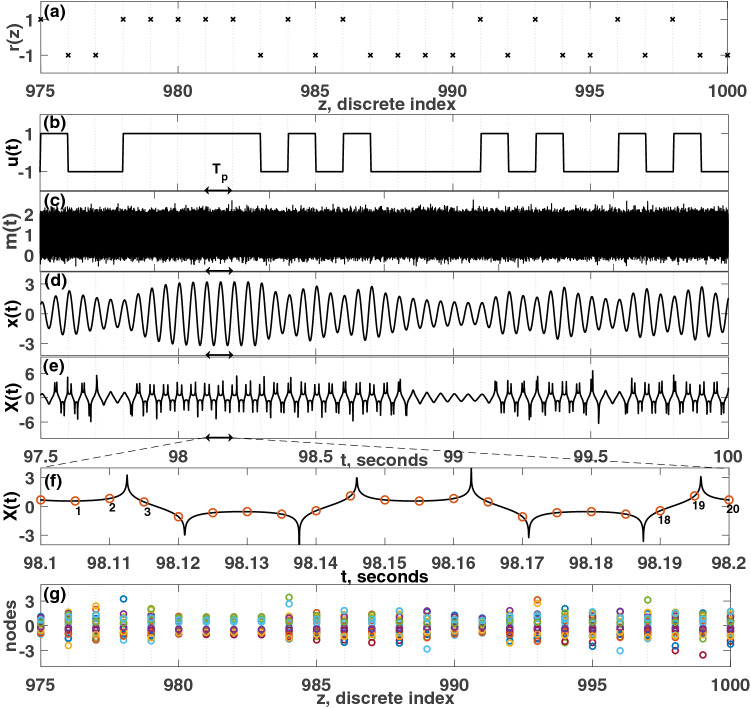
(**a**) Discrete random binary signal, *r*(*z*). (**b**) Continuous input signal, *u*(*t*). (**c**) Stochastic masking function, *m*(*t*). (**d**) Time history of *x*(*t*). (**e**) Rescaled time history, *X*(*t*). (**f**) 20 equidistant nodes for a single pseudo-period, $T_p$, are denoted with circles. (**g**) Collected nodal states from the nodes for machine learning input data set. Different colors in (**g**) denote different nodes. For the simulation depicted here, the parameters were set such that: $\mu =5$, $A=0.5$, $\Omega =40\pi$ rad/s, $\omega _0=40\pi$ rad/s, $T_p=0.1$ seconds, $N=20$ nodes, $\phi =\pi /3$ rad, $\sigma =100$, $\beta =1.0$.

For this task, the binary “false” and “true” values are encoded as discrete negative ones and positive ones, respectively, in a discrete signal, *r*(*z*). *r*(*z*) is defined such that $z \in {\mathbf {Z}}^+$ and $r(z) \in \{-1,+1\}$, which is depicted in Fig. 1a. To input this into a continuous dynamical system, these values are first mapped to a continuous input function, *u*(*t*), as follows:

5 $$\begin{aligned} u(t) = r(z) {\text { for }} (n-1)T_p \le t < (n)T_p{\text {, }}n \in {\mathbf {Z}}^+ \end{aligned}$$

This function is depicted in Fig. 1b. $T_p$ is a constant pseudo-period, in which *u*(*t*) does not change its value. Thus, for the XOR logical task, the input function, $u(t) \in \{-1,+1\}$, is a random square wave with a pseudo-period, $T_p$. This implies that each of the “true” (e.g., +1) or “false” (e.g., −1) values affect the system for an amount of time, $T_p$. The mask function, *m*(*t*), is depicted in Fig. 1c.

The Hopf PRC system described in Eq. (4) is numerically integrated using the Euler–Maruyama (EM) method, since the PRC is stochastic^42,47^. For these simulations, the integration time step, $dt=10^{-5}$ seconds, the total simulation time in this case was $3000 T_p=300$ seconds, and $T_p=0.1$ sec. This example simulation is shown in Fig. 1.

The time history of the *x* state obtained from the simulation is depicted in Fig. 1d. Next, *x*(*t*) is re-scaled by subtracting the mean, $\mu _x$, and dividing by the standard deviation, $\sigma _x$, using Eq. (6):

6 $$\begin{aligned} \begin{array}{ll} X&= Re\Big (tanh^{-1}(\frac{x-\mu _x}{\sigma _x})\Big ) \end{array} \end{aligned}$$

In this equation, the inverse hyperbolic tangent function is used as a nonlinear activation function. Only the real part of $tanh^{-1}(\frac{x-\mu _x}{\sigma _x})$ is used for the subsequent steps. The time history of the *X* state is depicted in Fig. 1e.

Next, equidistant nodes are created by dividing each pseudo-period, $T_p$, equally into $N(=20)$ nodes, as shown in Fig. 1f. Over each pseudo-period, $T_p$, the *N* node values are referred to as the nodal state, which is depicted in Fig. 1g.

**Figure 2:**
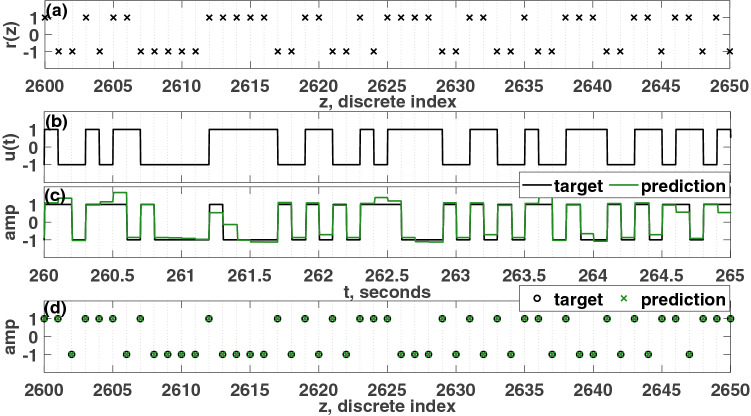
(**a**) Discrete random binary signal, *r*(*z*). (**b**) Continuous input signal, *u*(*t*). (**c**) target signal and continuous prediction. d) Discretized target and prediction. The calculated information metric is $R=0.98$. For the simulation depicted here, the parameters were set such that: $\mu =5$, $A=0.5$, $\Omega =40\pi$ rad/s, $\omega _0=40\pi$ rad/s, $T_p=0.1$ s, $N=20$, $\phi =\pi /3$ rad, $\sigma =100$, $\beta =1.0$.

The node matrix, *S*, is an $N \times K$ matrix; for this example, $N=20$ is the number of nodes over a pseudo-period, and $K=3000$ is the total number of pseudo-periods. Truncating the final 20% of this *S* matrix ($600T_p$), a new matrix, *L* ($480T_p$) is formed, which will be used in the training process. Throughout this paper, next, the reservoir computer is trained using ridge regression, as in Eq. (7):

7 $$\begin{aligned} \begin{array}{ll} w &{} = M L^{T}(L L^{T}+\lambda I)^{-1}\\ o(k) &{} = \sum _{i=1}^{N} w_i X_i(k) \end{array} \end{aligned}$$

A target signal (the *M* vector) is created from the encoded input based on a benchmark task, which in this case is XOR task. For each pseudo-period, there will be one target value that is found by performing the XOR operation between the inputs, *r*(*z*) and $r(z-1)$. In this way, the target vector, *M*, is found for the XOR task. Linear regression based training is then applied to the nodal state matrix, *L*, to map it to the desired output using Eq. (7). In Eq. (7), *w* is the weight vector found after training, *I* is the identity matrix, $\lambda =10^{-1}$ is the regularization parameter used to avoid over-fitting, and *o*(*k*) is the prediction of the reservoir computer at the *k*th pseudo-period. The discrete input, r(z) and continuous input, u(t) are given in Fig. 2a and b respectively. Figure 2c shows this prediction along with the corresponding target signal. In the final step, the prediction is binarized since XOR is a binary task, which is depicted in Fig. 2d. It should be noted that a nonlinear dynamic emulation task would not require this final step of discretization.

For a logical task, the efficacy of the reservoir computer is quantified using Shannon’s *information rate*^48^. The information rate, *R*, can be defined as follows:

8 $$\begin{aligned} R=H(x)-H_y(x) \end{aligned}$$

Here *H*(*x*) is the *Shannon entropy*, which denotes how much information is encoded in a signal. This can be defined as follows:

9 $$\begin{aligned} H(x)=-\sum _{i} p_i \log _2(p_i) \end{aligned}$$

In this equation, $p_i$ is the probability of getting a particular bit, *i*. $H_y(x)$ is the *conditional entropy*, which denotes the probability of getting an incorrect bit in the target signal:

10 $$\begin{aligned} H_y(x)=-\sum _{i,j} p(i,j) \log _2(p_i(j)) \end{aligned}$$

Here $p_i(j)=p(j|i)=\frac{p(i,j)}{\sum _j p(i,j)}$ and *p*(*i*, *j*) is the joint probability distribution of the two variables, *i* and *j*, each of which can take a value of “1” or “− 1” for a logical task. *i* is a bit from the target, and *j* is a bit from the prediction. The information rate, *R*, for this case was calculated to be 0.98 based on the prediction from the validation portion (not including in the training process). Due to the nature of this binary target signal, the Shannon entropy is 1.0, which marks the maximum value of the information rate for this task. It should be noted that the lower limit of *R* is zero, which would be achieved if every prediction was incorrect, while the upper limit of *R* depends on the task. For the parity tasks considered here, the upper limit of *R* is equal to one.

## Pseudo-period and noise

In this section, the effects of pseudo-period and noise on the computational ability of the reservoir are explored. For this discussion, several parity tasks (defined in Eq. (12)) are used to understand the effects of the pseudo-period and noise on the computational ability of the reservoir.

**Figure 3:**
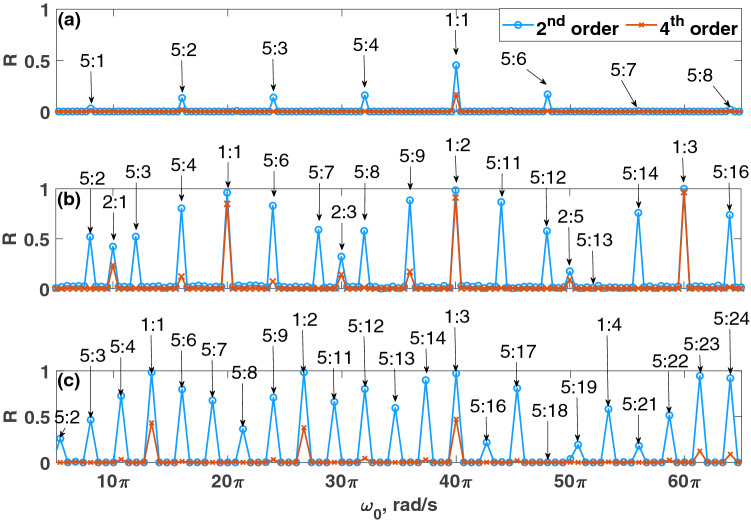
Comparison of the reservoir’s computing performance, *R*, on the choice of the pseudo-period, $T_p$, and the natural frequency, $\omega _0$ using 2nd and 4th order parity tasks. (**a**) $T_p=0.05$ seconds, (**b**) $T_p=0.1$ seconds, and (**c**) $T_p=0.15$ seconds. Different ratios of the natural period and pseudo-period (e.g., $\frac{2\pi }{\omega _0}:T_p$) are simulated, and the ratios are depicted for peaks in the information metric. $\omega _0=\Omega =50\pi$ rad/s is the resonance case. Parameters were set such that: $\mu =5$, $A=0.5$, $\Omega =50\pi$ rad/s, $N=1000$ nodes, $\phi =\pi /3$ rad, $\sigma =15$, $\beta =1.0$.

The relationship between the pseudo-period, $T_p$, and the natural frequency of the oscillator, $\omega _0$, is explored in Fig. 3 by using the 2nd and 4th order parity tasks. In Fig. 3a–c, the reservoir computer’s performance is measured for three different values of $T_p$ while varying the natural period, $\frac{2 \pi }{\omega _0}$. It is found that the reservoir has better performance when the pseudo-period is an integer multiple of the natural period of the oscillator. The reservoir’s performance is studied using both resonance ($\omega _0 = \Omega$) and non-resonance ($\omega _0\ne \Omega$) conditions. It is found that both cases can result in strong or weak computational ability depending on the fractional relationship between the natural period and the pseudo-period. However, maintaining this design can still fail to make a robust reservoir computer when $T_p$ is very low (e.g., $T_p=0.05$ seconds). For the remainder of the paper, combinations of $T_p$ and $\omega _0$ are chosen such that the pseudo-period is an integer multiple of the natural period of the oscillator.

**Figure 4:**
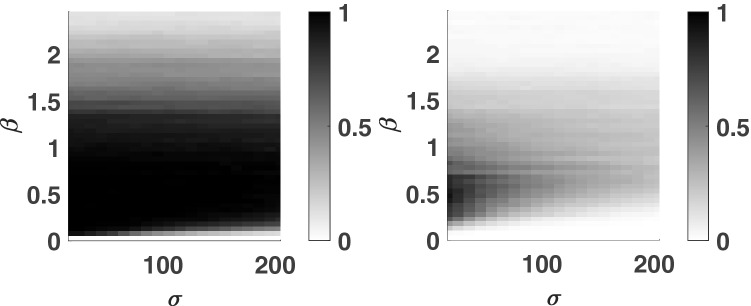
The reservoir computer is somewhat robust to noise. The effects of $\sigma$ and $\beta$ are shown. Left: 4th order parity task. Right: 6th order parity task. Parameters were set such that: $\mu =5$, $A=0.5$, $\Omega =40\pi$ rad/s, $\omega _0=40\pi$ rad/s, $T_p=0.1$ seconds, $N=1000$ nodes, and $\phi =\pi /3$ rad.

Noise is ubiquitous in physical systems. For this reason, noise is introduced into this system using a stochastic masking function. Figure 4 shows the relationship between the computational ability, as measured with *R*, the noise amplitude, $\sigma$, and the noise bias, β. The simulations presented in Fig. 4 are performed for the 4th order parity task (left) and 6th order parity task (right). The reservoir is found to be robust against a certain level of noise intensity, which demonstrates its potential to be implemented under the influence of environmental noise. However, increasing noise intensity does decrease the computational ability of the reservoir. This effect may be observed for a higher order task, which requires a longer memory (e.g., the 6th order parity task of Fig. 4). When $\beta =0$, the computational ability was the lowest. Since the non-periodic noise mask with increasing noise intensity deteriorates the computational ability, it should be noted that the Hopf reservoir computer can also be built by excluding the noise mask ($\sigma =0$).

## Analog circuit experiment

To build a physical reservoir computer (PRC), an analog circuit implementation of Eq. (4) was designed, fabricated, and tested. The circuit’s equations are given in Eq. (11):

11 $$\begin{aligned} \begin{array}{ll} {\dot{V}}_x = &{} -\frac{1}{R_1 C} \Big ( V_\mu (1+V_u V_m) -(V_x^2+V_y^2) \Big ) V_x + \frac{1}{R_1 C} V_{\omega _0} V_y - \frac{1}{R_2 C} \big ( A(1+V_u V_m) \sin (\Omega t+\phi ) \big ) \\ {\dot{V}}_y = &{} -\frac{1}{R_1 C} \Big (V_\mu (1+V_u V_m) -(V_x^2+V_y^2) \Big ) V_y - \frac{1}{R_1 C} V_{\omega _0} V_x \end{array} \end{aligned}$$

**Figure 5:**
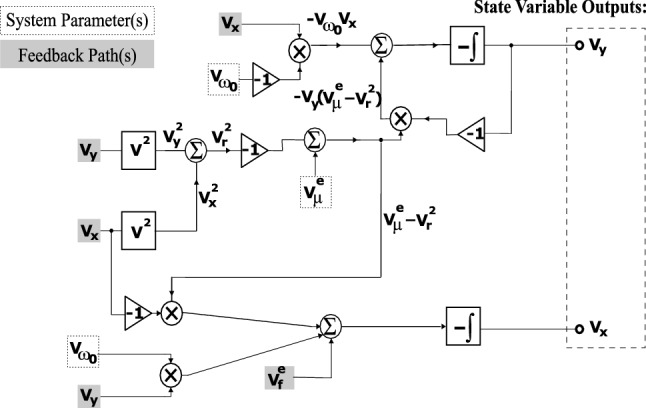
A simplified schematic for the Hopf PRC, with states $V_x$ and $V_y$. $V^e_\mu =V_\mu (1+V_u V_m)$ and $V^e_f= A(1+V_u V_m) \sin (\Omega t+\phi )$.

Here, $V_u$ is the input voltage, $V_m$ is the stochastic masking voltage, $V_\mu$ is the limit cycle radius voltage, $V_{\omega _0}$ is the resonance constant voltage. $V_x$ and $V_y$ are the states, which correspond to states *x* and *y* in Eq. (4). The circuit implementation used TL082 operational amplifiers and AD633 multipliers in standard integrator network configurations. The error tolerance is 1% for the resistors and $2\%$ for the capacitors. The continuous input function, $V_u$, the stochastic masking function, $V_m$, and the sinusoidal forcing, $\sin (\Omega t+\phi )$, were created in MATLAB and sent to the circuit via a National Instrument (NI) cDAQ-9174. This cDAQ-9174 also collected the $V_x$ and $V_y$ states. A sampling frequency of $10^5$ samples/s was used to collect data for all the experiments. The resistor values were chosen such that $R_1=10$ k$\Omega$ and $R_2=100$k$\Omega$, and the capacitor values were chosen such that $C=0.1\mu$F. A simplified schematic is shown in Fig. 5.

**Figure 6:**
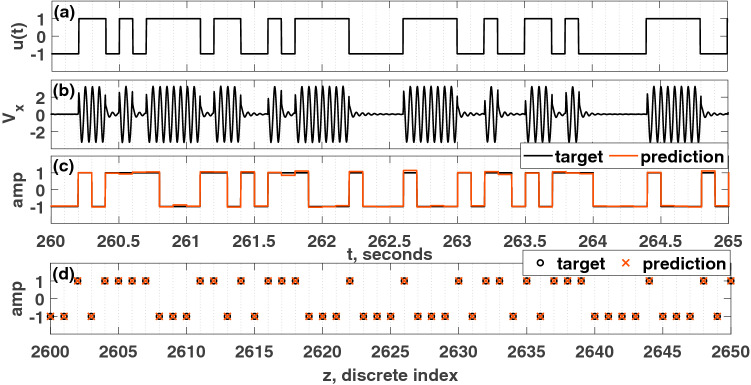
(**a**) Input voltage signal, $V_u$. (**b**) Time history of $V_x$. (**c**) XOR target signal, *M*, and the prediction. (**d**) Discretized prediction. The calculated information metric is $R=1.0$. For the experimental results depicted here, the parameters were set such that: $V_\mu =5$ volts, $A=0.5$ volts, $\Omega =40\pi$ rad/s, $V_{\omega _0}=40\pi$ volts, $T_p=0.1$ seconds, $N=20$ nodes, $\phi =\pi /3$ rad, $\sigma =10$ volts, $\beta =1.0$ volts.

The $V_x$ state will be treated in the same manner that the *x* state was treated in “Mapping methodology” section. That is, the $V_x$ state will be rescaled using Eq. (6), and then the rescaled state will be used to form the nodal state matrix, *L*. The target signal vector, *M*, will be created following the same process discussed in “Mapping methodology” section. Finally, Eq. (7) will be used to train the PRC to map input data to the desired output values. As an example, the analog circuit Hopf PRC was used to solve the XOR task as in the previous section, which is depicted in Fig. 6. The information rate, *R*, for this case was calculated to be 1.0 based on the prediction from the validation portion (not including in the training process).

## Benchmark tasks for Hopf PRC

The Hopf PRC is numerically and experimentally tested with three benchmark tasks: (1) *logic tasks*, (2) *emulation tasks of time series*, and (3) * prediction tasks*. Logic tasks include the fundamental logic gate tasks and parity tasks of different orders. Emulation tasks of time series will test the PRC’s ability to reproduce nonlinear auto regressive moving average (NARMA) tasks of different orders. Prediction tasks include the Santa Fe time series and sunspot prediction tasks.

### Logic benchmark tasks

#### Parity tasks

The computing efficacy of the reservoir is first evaluated with parity benchmark tasks. Since it is a logical task, the input function, *u*(*t*), is generated with a random binary signal, *r*(*z*), as discussed in “Mapping methodology” section. The *n*th order parity function, $P_n$, is defined by the following equation:

12 $$\begin{aligned} \begin{array}{ll} P_n(t)&=\prod \limits _{i=0}^{n-1}u(t-i T_p) \end{array} \end{aligned}$$

**Figure 7:**
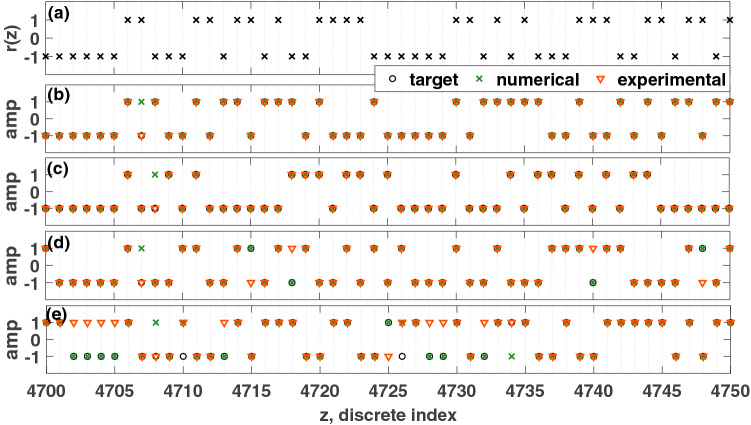
Comparison of the performance of the PRC for parity tasks. (**a**) Discrete input function, *r*(*z*). (**b**) 2nd order parity task. Information metric: $R_{exp}=1.00$, $R_{sim}=0.98$. (**c**) 3rd order parity task. Information metric: $R_{exp}=1.00$, $R_{sim}=0.98$. (**d**) 4th order parity task. Information metric: $R_{exp}=0.68$, $R_{sim}=0.93$. (**e**) 5th order parity task. Information metric: $R_{exp}=0.31$, $R_{sim}=0.74$. Parameters were set such that: $V_\mu =\mu =5$, $A=0.5$, $\Omega =40\pi$ rad/s, $V_{\omega _0}=\omega _0=40\pi$, $T_p=0.1$ seconds, $N=1000$ nodes, $\phi =\pi /3$ rad, $\sigma =15$, $\beta =1.0$, and a total time of $5000T_p=500$ seconds (only a portion of the discrete prediction is shown).

As *n* increases, this task will require more memory and nonlinearity from the reservoir. As given in “Mapping methodology” section, Shannon’s information metric is used to measure the performance of the PRC for logic tasks. For $n=1$, the first-order task does not require any memory from the input of the previous pseudo-period, so the task is linear. For $n>1$, the task is nonlinear, which demands that the reservoir computer must also possess memory and the nonlinear separation ability. In Fig. 7, the ability of the Hopf PRC to follow parity tasks of 2nd to 5th order, both experimentally and in simulations. The initial $4000T_p=400$ seconds are used for training, and the final $1000T_p=100$ seconds are used for testing. The performance difference between the PRC experiment and the simulation could be due to the presence of nonlinear circuit components in the analog circuit, which are not represented in Eq. (11). For instance, $V_u$ must jump between $-1$ and $+1$, but this instantaneous change takes a finite amount of time in the circuit.

#### Fundamental logic gate tasks

**Figure 8:**
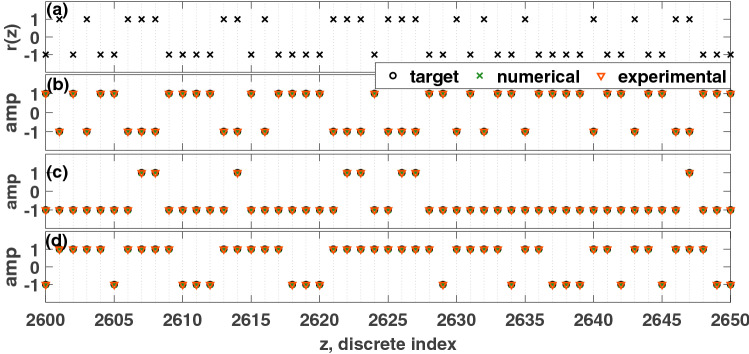
Comparison of the performance of the PRC for parity tasks. (**a**) Input function, *u*(*t*). (**b**) NOT ($\lnot$) gate. (**c**) AND ($\wedge$) gate, (**d**) OR ($\vee$) gate. For all numerical and experimental results, the information rate was at the theoretical maximum; the Hopf PRC can act as any of the fundamental logic gates. Parameters were set such that: $V_\mu =\mu =5$, $A=0.5$, $\Omega =40\pi$ rad/s, $V_{\omega _0}=\omega _0=40\pi$ rad/s, $T_p=0.1$ seconds, $N=1000$ nodes, $\phi =\pi /3$ rad, $\sigma =15$, $\beta =1.0$, and a total time of $3000T_p=300$ seconds. Only a portion of the response is shown here.

The computing performance of the reservoir is also assessed with fundamental logic gates: NOT ($\lnot$), AND ($\wedge$), and OR ($\vee$). The input function, *u*(*t*), is generated with a random binary signal as discussed in “Mapping methodology” section, and the Shannon’s information metric is used again to measure the performance of this PRC. Figure 8 depicts the response of the Hopf PRC acting as fundamental logic gates, both experimentally and in simulations. In all cases, the Hopf PRC achieved an information rate that was maximal.

### Emulation tasks

The reservoir is also evaluated with emulation tasks. The *nonlinear auto-regressive moving average* (NARMA) time series is used to test whether the reservoir possesses adequate nonlinearity and long time lags^4,24,26,28^. These tasks show the multi-tasking capability of the reservoir. NARMA tasks from the 2nd to 20th orders are used to test the reservoir. A NARMA task of order *n* is given in Eq. (13), where the initial target values are set to 0.19:

13 $$\begin{aligned} \begin{array}{ll} M_2(j+1) &{} = 0.4 M_2(j)+0.4 M_2(j) M_2(j-1)+0.6u^3(j\Delta t)+0.1\\ M_n(j+1) &{} = \alpha M_{n}(j)+\zeta M_{n}(j) \bigg (\sum \limits _{i=0}^{n-1} M_{n}(j-i) \bigg )+\gamma u\Big (\big (j-(n-1) \big ) \Delta t \Big ) u(j \Delta t)+\delta \\ u(t) &{} =0.2 \sin {\big (2\pi f_1 t\big )} \sin {\big (2\pi f_2 t\big )} \sin {\big (2\pi f_3 t\big )} \end{array} \end{aligned}$$

In Eq. (13), $M_n$ is the target of the system. *n* is the order of NARMA task, $(f_1,f_2,f_3) = (\frac{2.11}{500}, \frac{3.73}{500}, \frac{4.33}{500})$, and $(\alpha ,\zeta ,\gamma ,\delta )=(0.3, 0.05, 1.5, 0.1)$^26,28^. *u*(*t*) is the continuous input that is used to force the Hopf PRC, which is a function of a three sinusoidal functions. It should be noted that this formulation of the NARMA emulation task is non-standard. The *u*(*t*) given in Eq. (13) was used for other dynamic systems in which inertia played a large role^4,26^. Similarly, this non-standard NARMA task is used here to evaluate this analog circuit reservoir. The reservoir emulates this nonlinear function, but it should be noted that the correlation present in Eq. (13) does not allow a definitive evaluation of the long-term memory characteristics of this reservoir.

**Figure 9:**
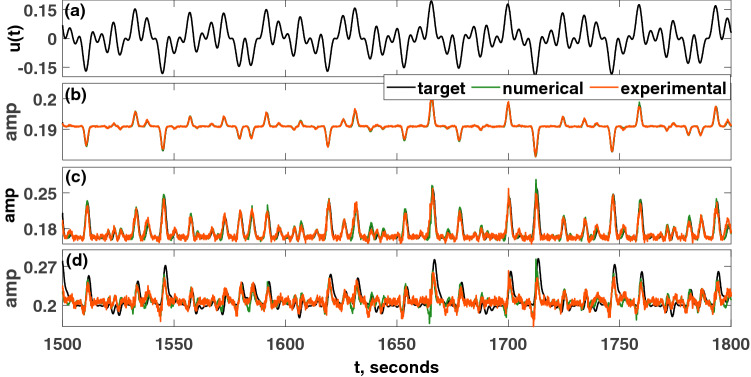
Comparison of the performance of the PRC for NARMA tasks. (**a**) Input function, *u*(*t*). (**b**) 2nd order NARMA task. Performance metric: $NMSE_{exp}=2.8181\times 10^{-6}$, $NMSE_{sim}=7.8199\times 10^{-7}$. (**c**) 10th order NARMA task. $NMSE_{exp}=0.0037$, $NMSE_{sim}=5.0362\times 10^{-4}$. (**d**) 20th order NARMA task. $NMSE_{exp}=0.0060$, $NMSE_{sim}=0.0033$. Only a portion of the result is shown in each figure. Parameters were set such that: $V_\mu =\mu =5$, $A=0.5$, $\Omega =40\pi$ rad/s, $V_{\omega _0}=\omega _0=40\pi$, $T_p=0.1$ seconds, $N=1000$ nodes, $\phi =\pi /3$ rad, $\sigma =15$, $\beta =1.0$.

In the simulations and experiments, $\Delta t=0.1$ seconds, and the sampling rate was $10^5$ samples/second. Figure 9 shows several NARMA tasks. Instead of the information rate, the *normalised mean square error* (NMSE) is used to evaluate the performance of the reservoir computer for the NARMA tasks:

14 $$\begin{aligned} NMSE=\frac{\sum \nolimits _{j=j_0}^{j_{f}}\Big (M_{n}(j+1)-o_n(j+1)\Big )^2}{\sum \nolimits _{j=j_0}^{j_f}M^2_{n}(j+1)} \end{aligned}$$

**Figure 10:**
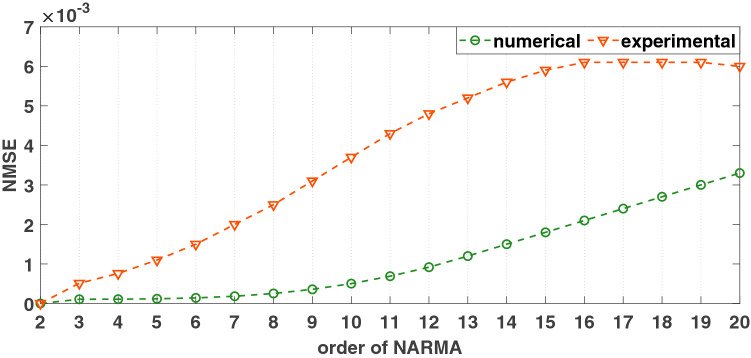
Plot of the NMSE of the 2nd to 20th order NARMA tasks for the simulation and experiment. Parameters were set such that: $V_\mu =\mu =5$, $A=0.5$, $\Omega =40\pi$ rad/s, $V_{\omega _0}=\omega _0=40\pi$, $T_p=0.1$ seconds, $N=1000$, $\phi =\pi /3$ rad, $\sigma =15$, $\beta =1.0$.

The final 20% of the target signal (16,000–20,000 pseudo-periods) is used for the validation. $M_n$ is the target, and $o_n$ is the prediction from the reservoir computer. In Eq. (14), $j_0$ is the starting time step, and $j_f$ is the ending time step from the test section. From the plots in Figs. 9 and 10, the numerical simulations of the Hopf PRC show superior performance as compared to the experiment. However, both have an acceptable performance until the 20th order task. The PRC can perform much higher order NARMA tasks than the order of the parity tasks.

### Prediction tasks

#### Santa Fe task

**Figure 11:**
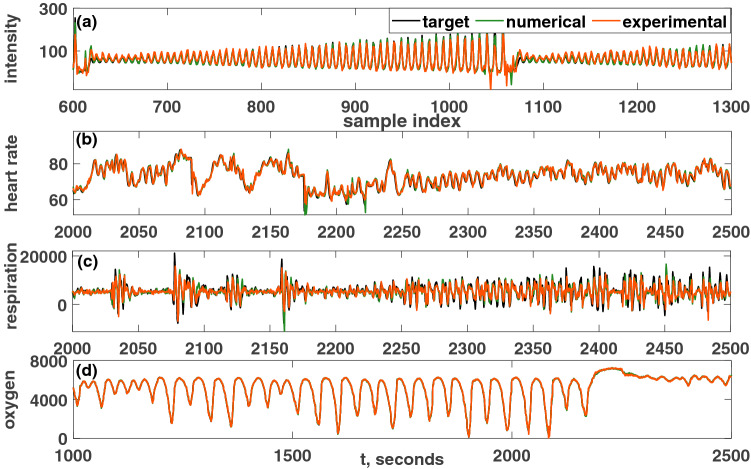
Comparison of the performance of the PRC for Santa Fe prediction tasks. (**a**) The Santa Fe chaotic time series of a laser intensity prediction task. Performance metric: $NMSE_{exp}=0.0615$, $NMSE_{sim}=0.02$. (**b**) The Santa Fe heart rate prediction task. $NMSE_{exp}=6.0258\times 10^{-4}$, $NMSE_{sim}=6.5060\times 10^{-4}$. (**c**) Santa Fe respiration force prediction task. $NMSE_{exp}=0.1826$, $NMSE_{sim}=0.1753$. (**d**) Santa Fe blood oxygen concentration prediction task. $NMSE_{exp}=3.3287\times 10^{-4}$, $NMSE_{sim}=1.7\times 10^{-4}$. Parameters were set such that: $V_\mu =\mu =5$ volts, $A=0.5$ volts, $\Omega =40\pi$ rad/s, $V_{\omega _0}=\omega _0=40\pi$ volts, $T_p=0.1$ seconds, $N=1000$ nodes, $\phi =\pi /3$ rad, $\sigma =15$ volts, $\beta =1.0$ volts. Only a portion of the result is shown in each figure.

Time series forecasting is an important benchmark for a reservoir. The Santa Fe time series was first used in a time series forecasting competition as a benchmark test. The Santa Fe time series data set *A* is a univariate time series found from the recorded intensity of a chaotic far-infrared-laser^49^. The target signal is generated to predict the value at the next time step based on the values of the current and previous time steps. Figure 11a shows the Hopf PRC’s performance on this laser time series, for both the experiment and the numerical simulations. NMSE is used as the performance metric.

Santa Fe time series data set *B* is a multivariate time series found from the sleep laboratory of the Beth Israel Hospital (current name: Beth Israel Deaconess Medical Center) in Boston, Massachusetts^50,51^. This data set was taken from the MIT-BIH Polysomnographic Database record (slp60) and submitted to the Santa Fe Time Series Competition in 1991^52^. The heart rate, chest volume (respiration force), and blood oxygen concentration comprise the target.

For each of these time series, the target signal is again generated to predict the next step based on the values of the current and previous time steps. In each case, the original time series is normalized to use as the input. Figure 11b–d shows the reservoir computer’s performance in predicting subsequent values of the heart rate, respiratory force, and blood oxygen concentration, respectively, through both experiments and numerical simulations. The NMSE is calculated in each case to evaluate the performance of the reservoir.

#### Sunspot prediction task

**Figure 12:**
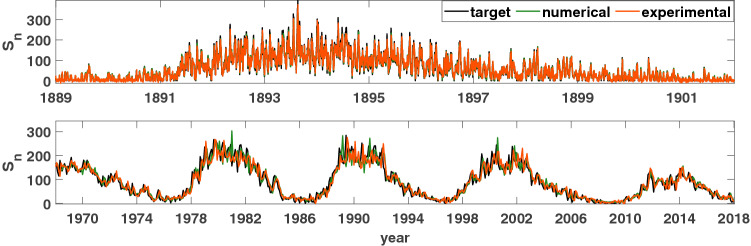
Comparison of the performance the sunspot prediction ($S_n$) task. Top: Daily total number of sunspot prediction task, for both the experiment and the numerical simulations. Performance metric: $NMSE_{exp}=0.0548$, $NMSE_{sim}=0.0534$. Bottom: Monthly mean total number of sunspot prediction task. Performance metric: $NMSE_{exp}=0.0595$, $NMSE_{sim}=0.0455$. Parameters were set such that: $V_\mu =\mu =5$ volts, $A=0.5$ volts, $\Omega =40\pi$ rad/s, $V_{\omega _0}=\omega _0=40\pi$ rad/s, $N=1000$ nodes, $\phi =\pi /3$ rad, $\sigma =15$ volts, $\beta =1.0$ volts. Only a portion of the result is shown in each figure.

The prediction of the total number of sunspots ($S_n$) is also a one-step time series prediction task similar to the Santa Fe time series^10^. Daily and monthly total sunspot numbers were used in one step forecasting purpose by the reservoir computer. The necessary data set is taken from *WDC-SILSO*, Royal Observatory of Belgium, Brussels^53^. Again, for each of the time series, the target signal is generated to predict the next value based on the value of the current and previous time steps, and the original time series is normalized to use as the input to the oscillator. Figure 12 (top) shows the reservoir’s performance in predicting the next steps of the daily total counted sunspots, and Fig. 12 (bottom) shows the performance in predicting monthly counted sunspots. Again, the NMSE is used to evaluate the reservoir’s efficacy for this task.

## Concluding remarks

In this paper, the Hopf oscillator is explored as a physical reservoir computer through employing a time-multiplexed, node-based architecture with a stochastic masking function. Discarding the regularly used delay lines, this Hopf PRC is a simple and cheap method for creating a physical reservoir computer. Since quantum systems are capable of limit cycle motion^54^, this Hopf PRC formulation might be applicable for quantum PRCs. The Euler–Maruyama method was used for the numerical simulations of this Hopf PRC. An analog circuit of this Hopf PRC was developed, fabricated, and tested. The Hopf PRC was found to possess multi-tasking capability, since it was shown to perform logic operations, emulation tasks, and time series prediction tasks. Taking inspiration from adaptive oscillators, the input signal was injected into multiple locations, including the parameter that affects the limit cycle radius and the amplitude of the sinusoidal forcing. Additionally, the masking function used in this PRC is stochastic. Since this PRC architecture is tested with noise, it also suggests that this reservoir computer should be robust to environmental noises in practical implementations.
